# Why a Confirmation Strategy Dominates Psychological Science

**DOI:** 10.1371/journal.pone.0138197

**Published:** 2015-09-18

**Authors:** David M. Sanbonmatsu, Steven S. Posavac, Arwen A. Behrends, Shannon M. Moore, Bert N. Uchino

**Affiliations:** 1 Department of Psychology, University of Utah; Salt Lake City, Utah, United States of America; 2 Owen Graduate School of Management, Vanderbilt University, Nashville, Tennessee, United States of America; Tilburg University, NETHERLANDS

## Abstract

Our research explored the incidence and appropriateness of the much-maligned confirmatory approach to testing scientific hypotheses. Psychological scientists completed a survey about their research goals and strategies. The most frequently reported goal is to test the non-absolute hypothesis that a particular relation exists in some conditions. As expected, few scientists reported testing universal hypotheses. Most indicated an inclination to use a confirmation strategy to test the non-absolute hypotheses that a particular relation sometimes occurs or sometimes does not occur, and a disconfirmation strategy to test the absolute hypotheses that a particular relation always occurs or never occurs. The confirmatory search that dominates the field was found to be associated with the testing of non-absolute hypotheses. Our analysis indicates that a confirmatory approach is the normatively correct test of the non-absolute hypotheses that are the starting point of most studies. It also suggests that the strategy of falsification that was once proposed by Popper is generally incorrect given the infrequency of tests of universal hypotheses.

## Introduction

One of the enduring legacies of Karl Popper’s [1] philosophy of science is his belief in the central role of falsification in scientific advancement [2,3]. According to Popper, scientific theories can never be conclusively verified. Although evidence may be gathered which is consistent with a theory, the possibility always remains that instances will be uncovered that prove it to be false. In contrast, scientific generalizations can be conclusively falsified by a single disconfirming observation. Thus, science progresses primarily through falsification. Negative evidence permits the rejection of erroneous theories and allows the promotion of more viable alternatives.

Popper’s [1] provocative analysis of how science progresses is unsettling, because it is largely inconsistent with what psychological scientists do and have always done. Rather than following a strategy of falsification, most researchers attempt to provide confirming evidence for their hypotheses. Evidence of the approach taken by most psychological scientists was provided by Uchino, Thoman, and Byerly [4], who analyzed papers published in the *Journal of Personality and Social Psychology* over a 23 year period. An examination of the studies reported in a large sample of the journal’s papers showed that the vast majority (76.7%) took a confirmatory approach involving the testing of a favored hypothesis. Almost all of the reported studies (91.3%) supported an existing theory. Only 21.6% discussed alternative hypotheses and only 11.4% mentioned testing competing hypotheses. Thus, a strategy of falsification and its epistemological cousin, the crucial testing of alternative theories [5–7], appear to be atypical of psychology. This suggests that someone must be wrong; either scientists are going about their business incorrectly or Popper was mistaken about how science progresses.

### Everyday Hypothesis Testing

The approach taken by psychological scientists is similar to how people generally test their ideas. Research has shown that in the selective testing of hypotheses [8], people typically engage in a positive or confirmatory search for instances of the presumed relation between variables [9,10]. They may also assimilate the gathered evidence in a manner that is consistent with the hypothesis or expectations [8,11]. Nevertheless, the search for confirming evidence does not necessarily lead to verification. Instead, the hypothesis is often falsified if instances are discovered that do not hold true in the predicted conditions [12,13].

Although confirmation appears to be the default strategy in the everyday testing of hypotheses [14], there are important conditions in which a disconfirmatory approach predominates. Sanbonmatsu, Posavac, Vanous, and Ho [15] have shown that the search for evidence depends heavily on the hypothesized frequency of the test relation. Hypotheses specify in general terms the proportion of instances that are characterized by a particular pattern, relation, or effect. At the broadest level, hypotheses presume that a phenomenon is either present or absent. Additionally, they are either absolute in presuming that a particular relation is always present or always absent, or non-absolute in presuming that a relation is sometimes present or sometimes absent. The informativeness of confirming vs. disconfirming evidence depends on the hypothesized frequency of the test relation [14]. Broadly speaking, the diagnosticity of a piece of information or datum can be defined in terms of the degree to which it distinguishes the test hypothesis from its complement [12]. A datum is informative to the extent that the probability of the datum when the hypothesis is true differs from the probability of the datum when the hypothesis is not true. In tests of absolute or universal hypotheses, disconfirmations have considerably greater diagnostic value than confirmations. A confirming observation is probable or possible not only when an absolute hypothesis is true but also when it is false. In contrast, a disconfirming instance is not possible when an absolute hypothesis is true. This, of course, is in keeping with Popper’s [1] analysis of the utility of falsification in science. In contrast, confirmations are much more diagnostic than disconfirmations in tests of non-absolute hypotheses presuming that a test relation occurs in some instances. A disconfirming observation is probable not only when the non-absolute hypothesis is false but also when it is true. In contrast, a confirming observation is not possible when a non-absolute hypothesis is false.

Sanbonmatsu, et al. [15] investigated whether the likelihood of a confirmatory vs. disconfirmatory search varies as a function of the hypothesized frequency of the test relation. Across three studies, they found that participants tended to seek evidence disconfirming the hypotheses that a phenomenon always occurs or never occurs, and evidence confirming the non-absolute hypothesis that a phenomenon sometimes occurs. For example, participants in one study were given the task of testing whether a statement about a set of integers from 1 to 10 was true. Some tested the absolute hypothesis that all of the numbers were even or the absolute hypothesis that none of the numbers were even, while others tested the non-absolute hypothesis that some of the numbers were even. They generated individual numbers, one possible number at a time, to test whether they were members of the set. As expected, participants tended to take a disconfirmatory approach to testing absolute hypotheses. For example, they tested odd numbers such as 3 to assess the hypothesis that all of the numbers were even. In contrast, participants typically took a confirmatory approach to test the hypothesis that some of the numbers were even. That is, they tested whether even numbers such as 2 belonged to the set. The findings were in line with previous research showing that people seek the most diagnostic evidence in the testing of hypotheses [16,17]. More generally, the results were consistent with the conception of people as able and flexible thinkers who utilize different test strategies as a function of the context [18].

The findings of the Sanbonmatsu, et al. [15] study raise questions about why a confirmation strategy dominates psychological science. If scientists think as well as research participants and, hence, are sensitive to the diagnosticity of evidence in the testing of hypotheses, why do they generally take a confirmatory approach in their studies?

### Is Science Absolute?

Popper [1] assumed that scientific theories are universal; he believed they postulate that a phenomenon holds true in all conditions or instances. Of course, if theories are absolute, the most informative approach is the strategy of falsification that he prescribed. Again, when a phenomenon is hypothesized to always occur, a single negative observation is sufficient to reject the hypothesis while positive observations are inconclusive.

However, the ideas that are generated and tested in science may not always be absolute. To the contrary, we believe that most scientific hypotheses and theories predict that a particular relation between variables exists in some instances. For example, water turns to ice in some atmospheric conditions. Smoking contributes to lung cancer in some smokers. Anxiety sometimes undermines task performance. If the testing of non-absolute hypotheses is the most prevalent goal, the confirmatory approach that dominates psychological science [4] may be normatively correct. Note that when scientists hypothesize that a relation exists in some conditions, they presume that the relation exists uniformly in some conditions. That is, they believe there is invariance or regularity in nature [19] such that an effect that occurs in a particular context always occurs in that context (or nearly identical contexts).

### An Empirical Investigation of Scientific Goals and Strategies

The nature of the goals and approaches characterizing psychological research is an empirical question that is best addressed through systematic study. Unfortunately, most prior accounts of this important aspect of the scientific enterprise have been speculative and based heavily on informal observation. In our investigation, psychological scientists completed a survey about their research practices. They began by reporting the research goals guiding their studies. We were particularly interested in the prevalence of tests of absolute vs. non-absolute hypotheses. The scientists were also asked about the general approach they take in the early stages of research aimed at establishing a phenomenon and in the later stages aimed at determining the causes and scope. Finally, the scientists indicated the strategies they use to test different research goals. We were interested in whether they tend to take a disconfirmatory approach to test absolute hypotheses and a more confirmatory approach to test non-absolute hypotheses.

## Method

### Participants

The University of Utah Institutional Review Board approved the procedures and consent process for this study (IRB protocol #77072 “Research goals and strategies”). The survey was preceded by a consent cover letter that described the purpose and procedures of the study. Participants were informed that “by proceeding and responding to the questionnaire, you are giving your consent to participate.”

We expected large differences (an effect size of .50) in the approach taken to test different types of hypotheses based on our previous work examining the impact of quantifiers on information search [15]. A power analysis adopting an alpha of .05 (2-tailed) and power of 80% indicated that a sample of at least 33 scientists was needed to demonstrate within-subjects differences. Because participation was solicited en masse, we had limited control over the exact number of respondents.

Scientists at 6 research universities working as faculty in psychology departments or psychology programs, or who were trained as psychologists were recruited to participate in the study. At the end of the survey, respondents indicated the field of psychology in which they were trained. Their diverse psychological backgrounds are presented in Table 1. Altogether, 17 female and 26 male scientists responded to the survey. The survey data are available in S1 Dataset.

**Table 1:** Psychology background of survey respondents.

Field of Psychology	Frequency
Clinical	13
Cognitive	10
Developmental	1
Neuroscience	5
Personality	1
Quantitative	2
Social	8
Other	3

### Procedure

Potential respondents were solicited for participation in a study of “research goals and strategies” via email. They were presented with the following study description:

This research is concerned with the goals and approaches that guide scientific research. There has been a great deal of philosophizing about the strategies that scientists use to test their theories and hypotheses. However, there has been a paucity of empirical research on this topic. The main purpose of this study is to examine the type of hypotheses that guide scientific research and the strategies used by scientists to test them.

The survey was administered online using Qualtrics. The first set of questions on the survey began with the following instructions:

Research often begins with a hypothesis about the relation between two or more variables. For example, many studies begin with the hypothesis that a variable X is significantly correlated with a variable Y. In particular, the presumption may be that a variable X has the property Y or that changes in variable X cause changes in a variable Y.

Scientific studies may be driven by a number of different hypothesis testing goals. Please indicate the extent to which each of the following goals guides your research.

Participants were presented with four possible research goals:

The goal is to test the hypothesis that a particular relation exists in *all* conditions. That is, the goal is to show that a particular relation *always* occurs or is *always* present in nature.

The goal is to test the hypothesis that a particular relation exists in *some* conditions. That is, the goal is to show that a particular relation occurs *sometimes* or is present *some* of the time in nature.

The goal is to test the hypothesis that a particular relation does *not* exist in *some* conditions. That is, the goal is to show that a particular relation does *not* occur *sometimes* or is *not* present *some* of the time in nature.

The goal is to test the hypothesis that a particular relation does *no*t exist under any conditions. That is, the goal is to show that a particular relation *never* occurs or is *never* present in nature.

The scientists’ task was to indicate the extent to which each goal guided their research on a scale containing the following four possible responses: *a*. *Primary goal of my studies; b*. *Frequent goal of my studies; c*. *Infrequent goal of my studies; d*. *Not a goal of my studies*.

The scientists were then asked about the approach they take to achieve their research goals. The first question was “Which of the following approaches do you typically take in your studies?” Two response alternatives were presented:

a. I usually take a confirmatory approach in which I try to show that a particular relation occurs or exists in at least one set of conditions.
b. I usually take a disconfirmatory approach in which I try to show that a particular relation does not occur or exist in at least one set of conditions.

They were then asked “In which of these phases of a research program are you more apt to take a confirmatory approach in which you attempt to verify that a particular relation exists in at least one set of conditions?” This was followed by the question “In which of these phases of a research program are you more apt to take a disconfirmatory approach in which you attempt to verify that a particular relation does not exist in at least one set of conditions? In both questions, the following two alternatives were presented:

a. The early phases of a research program aimed at establishing that a particular relation or phenomenon occurs or exists.
b. The later phases of a research program aimed at delineating the generality of a phenomenon and explaining a phenomenon.

The final set of questions presented psychological scientists with four different hypothesis testing goals:

Imagine you have been given the task of testing the hypothesis is that a particular relation *always* exists in nature. That is, you need conduct a study to test the hypothesis that a particular relation *always* occurs or is present in *all* conditions.

Imagine you have been given the task of testing the hypothesis is that a particular relation *sometimes* exists in nature. That is, you need conduct a study to test the hypothesis that a particular relation occurs *sometimes* or is present in *some* conditions.

Imagine you have been given the task of testing the hypothesis is that a particular relation *sometimes* does *not* exist in nature. That is, you need conduct a study to test the hypothesis that a particular relation does *not* occur *sometimes* or is *not* present in *some* conditions.

Imagine you have been given the task of testing the hypothesis is that a particular relation *never* exists in nature. That is, you need conduct a study to test the hypothesis that a particular relation does *not* occur or is *not* present in *any* conditions.

For each hypothesis, they were asked “If you were to conduct a study to test the truth or falsity of this hypothesis, which of the following research approaches would you more apt to take?” The following two alternatives were presented:

a. I would attempt to provide a demonstration of the presumed relation. That is, I would conduct a study to show that the presumed relation occurs or is present in at least one set of conditions.
b. I would attempt to provide a demonstration of the presumed relation not occurring. That is, I would conduct a study to show that the presumed relation does *not* occur or is *not* present in at least one set of conditions.

Thus, the scientists were asked whether they would attempt to demonstrate the presence of a particular relation or the absence of a particular relation. The survey ended with questions about the respondent’s gender and psychological training. Note that participants had the option of not responding to any of the survey questions.

## Results

### Research Goals

A one way within-subjects analysis of variance (ANOVA) indicated that the four possible goals varied significantly in the extent to which they guided research, *F*(1,45) = 26.35, *p* < .001. The means (see Table 2) suggest that the scientists’ studies are guided most commonly by the goal of demonstrating that a relation sometimes occurs or exists; a total of 95.7% of the scientists indicated that it is the primary goal (12 or 26.1%) or a frequent goal of their studies (32 or 69.6%). Research appears to be infrequently guided by the goal of testing the absolute hypotheses that a relation always occurs or never occurs; 41.3% (19) indicated that demonstrating that a relation always exists is “not a goal of my studies” and 76.1% (35) indicated that demonstrating that a relation never exists is “not a goal of my studies”.

**Table 2:** Basic goals guiding the studies of psychological scientists.

Goal	Mean	SD
The goal is to test the hypothesis that a particular relation exists in *all* conditions. That is, the goal is to show that a particular relation *always* occurs or is *always* present in nature.	3.28	.69
The goal is to test the hypothesis that a particular relation exists in *some* conditions. That is, the goal is to show that a particular relation occurs *sometimes* or is present *some* of the time in nature.	1.78	.51
The goal is to test the hypothesis that a particular relation does *not* exist in *some* conditions. That is, the goal is to show that a particular relation does *not* occur *sometimes* or is *not* present *some* of the time in nature.	2.57	.75
The goal is to test the hypothesis that a particular relation does *no*t exist under any conditions. That is, the goal is to show that a particular relation *never* occurs or is *never* present in nature.	3.74	.49

Notes: N = 46. Judgments were made on a 4 point scale anchored by 1 = *Primary goal of my* studies and 4 = *Not a goal of my studies*

A planned comparison indicated that scientists were more likely to investigate the hypothesis that a relation sometimes exists than the hypothesis that a relation always exists, *t*(45) = 11.45, *p* < .001, *d* = 2.47, 95% CI: 1.24 to 1.76. They were also more likely to test the hypothesis that a relation sometimes does not exist than the hypothesis that a relation never exists, *t*(45) = 10.77, *p* < .001, *d* = 1.85, 95% CI: 0.95 to 1.39. Further analyses verified that the scientists were much more apt to test non-absolute hypotheses (that a relation sometimes exists or sometimes does not exist) than absolute hypotheses (that a relation always exists or never exists), *M* = 2.18 vs *M* = 3.51, *t*(45) = 14.74, *p* < .001, *d* = 2.93, 95% CI: 1.15 to 1.52. They were also more likely to attempt to demonstrate the presence of a relation (i.e., that a relation always or sometimes exists) than the absence of a relation (i.e., that a relation sometimes does not exist or never exists), *M* = 2.53 vs *M* = 3.16, *t*(45) = 6.54, *p* < .001, *d* = 1.34, 95% CI: -0.81 to -0.43. Finally, the scientists’ studies are guided more by the goal of demonstrating that a particular relation sometimes exists than the goal of demonstrating that a particular relation sometimes does not exist, *t*(45) = 6.52, *p* < .001, *d* = 1.23, 95% CI: -1.02 to -0.54.

### Research Approach

The scientists indicated whether they generally engage in a confirmatory or disconfirmatory search in their studies, and then reported the phases of research in which their approach tended to be confirmatory and disconfirmatory. When asked about their general research approach, 95.7% (44) of the participants indicated “I usually take a confirmatory approach in which I try to show that a particular relation occurs or exists in at least one set of conditions” while only 4.3% (2) indicated “I usually take a disconfirmatory approach in which I try to show that a particular relation does not occur or exist in at least one set of conditions.” A binomial test indicated that participants are more likely to engage in a confirmatory search than would be expected by chance, *p* < .001.

When asked about when they are most apt to take a confirmatory approach, 82.6% (38) of the participants indicated that they are most likely to attempt to confirm the test relation in “The early phases of a research program aimed at establishing that a particular relation or phenomenon occurs or exists” while only 17.4% (8) of the scientists are most apt to attempt to confirm a test relation in the “The later phases of a research program aimed at delineating the generality of a phenomenon and explaining a phenomenon.” A binomial test suggested that more participants take a confirmatory approach in the early phases of a research program than in the later phases, *p* < .001.

When asked about the research phase in which they were most apt to take a disconfirmatory approach, only 14.3% (6) indicated that they are most likely to attempt to disconfirm a test relation in the early phases aimed at establishing the existence or occurrence of a phenomenon while the vast majority (36 or 85.7%) of the scientists are most apt to attempt to disconfirm a test relation in the later phases aimed at delineating the generality of a phenomenon and explaining a phenomenon.” A binomial test indicated that more participants reported taking a disconfirmatory approach in the later phases of a research program vs. the early phases than would be expected by chance, *p* < .001.

A correlational analysis explored the relation between the research goals and strategies of the scientists. The goal of demonstrating that a particular relation sometimes exists was positively correlated with a confirmatory rather a disconfirmatory approach, *r*(45) = .30, *p* = .042. This correlation was actually quite surprising given the near absence of variability in both measures. The type of approach taken was not correlated with the goals of demonstrating that a relation always exists, *r*(45) = .07, *p* = .653, sometimes does not exist, *r*(45) = -.162, *p* = .281, or never exists, *r*(45) = .114, *p* = .449. The findings suggest that scientists are more likely to take a confirmatory approach in their studies if their goal is to demonstrate that particular phenomena sometimes exist in nature.

### Testing Research Hypotheses

The final set of questions examined how the research approach of scientists varies as a function of their goals. We were interested specifically in whether the tendency to engage in a confirmatory vs. disconfirmatory search depends on the type of hypothesis under investigation. When the test hypothesis is that a relation sometimes or always exists, a confirmatory approach entails an attempt to demonstrate the presence of the hypothesized relation. However, two of the research hypotheses concerned the absence of a relation–the possibility that a particular relation does not occur sometimes or does not occur at all. A confirmation of the absence of a relation is a demonstration of the relation not occurring while a disconfirmation of the absence of a relation is a demonstration of the relation occurring. Thus, when the test hypothesis was that a relation sometimes does not occur or never occurs, a response of “I would attempt to show that the presumed relation does not occur. . .” was coded as a confirmatory approach while a response of “I would attempt to show that the presumed relation occurs…” was coded as a disconfirmatory approach.

Table 3 presents the approach the scientists are inclined to take to test the different types of hypotheses. A series of binomial tests were used to determine whether the scientists are more inclined to engage in a confirmatory or disconfirmatory search in testing each of the different types of hypotheses. Participants are much more likely to utilize a disconfirmatory search than a confirmatory search to test the absolute hypothesis that a test relation always occurs, *p* < .001. More than 80% indicated they would attempt to disconfirm the hypothesis that a possible relation is always present. They are much more inclined to take a confirmatory rather than a disconfirmatory approach to test the non-absolute hypotheses that a test relation sometimes occurs, *p* < .001, or sometimes does not occur, *p* < .002. In fact, more than 90% reported they would attempt to confirm the hypothesis that a relation is sometimes present and almost 75% reported they would attempt to confirm the hypothesis that a relation is sometimes not present. Finally, they are almost all inclined to take a disconfirmatory approach to test the absolute hypothesis that a test relation never occurs, *p* < .001. More than 95% indicated they would seek to disconfirm the hypothesis that a possible relation is never present.

**Table 3:** Proportion of scientists taking a confirmatory vs. a disconfirmatory approach as a function of the type of hypothesis under investigation.

Goal	Confirmatory	Disconfirmatory
Hypothesis that a particular relation *always* occurs or is present in *all* conditions.	18.6% (8)	81.4% (35)
Hypothesis that a particular relation occurs *sometimes* or is present in *some* conditions.	90.7% (39)	9.3% (4)
Hypothesis that a particular relation does *not* occur *sometimes* or is *not* present in *some* conditions.	74.4% (32)	25.6% (11)
Hypothesis that a particular relation does *not* occur or is *not* present in *any* conditions.	4.7% (2)	95.3% (41)

Notes: N = 43.

A planned comparison revealed that participants are much more likely to take a confirmatory approach to test the hypothesis that a particular relation sometimes occurs than to test the hypothesis that a particular relation always occurs, *Χ* ^*2*^ (1, *N* = 86) = 45.09, *p* < .0001. They are also much more likely to use a confirmatory strategy in testing the hypothesis that a particular relation sometimes does not occur than in testing the hypothesis that a particular relation never occurs, *Χ* ^*2*^ (1, *N* = 86) = 43.78, *p* < .0001. Further analyses revealed that a confirmatory approach is much more likely to be used in testing non-absolute hypotheses (that a particular relation sometimes occurs or sometimes does not occur) than in testing absolute hypotheses (that a particular relation always occurs or never occurs), *Χ* ^*2*^ (1, *N* = 172) = 86.83, *p* < .0001. Finally, a confirmatory approach is more likely to be taken in tests of hypotheses about the presence of a test relation (that a relation always or sometimes occurs) than in tests of hypotheses about the absence of a relation (that a relation never occurs or sometimes does not occur), *Χ* ^*2*^ (1, *N* = 172) = 3.94, *p* = .047.

## Discussion

Many books and articles have speculated about hypothesis testing in science. Our study took a more empirical approach to this important topic by surveying psychological scientists about their goals and strategies. The most commonly reported aim is to test the non-absolute hypothesis that a particular relation between variables occurs or exists sometimes. As expected, few scientists reported testing universal hypotheses. They also indicated that they are more likely to strive to establish the presence than the absence of a phenomenon. Following Uchino et al. [4], scientists reported they generally use a confirmation strategy, especially in the early phases of a research program. When they do use a disconfirmation strategy, it tends to be in the later phases aimed at explicating the scope and causes of a phenomenon.

Researchers appear to be aware that the diagnosticity of different search strategies depends on the hypothesis under investigation. They are highly inclined to engage in a confirmatory search to test the non-absolute hypotheses that a phenomenon sometimes occurs or sometimes does not occur, and a disconfirmatory approach to test the absolute hypotheses that a phenomenon always or never occurs. This along with the correlational evidence showing the linkage between the testing of non-absolute hypotheses and confirmation suggests that psychological scientists generally take a confirmatory approach because their most common goal is to demonstrate that a particular phenomenon sometimes exists in nature.

In order to increase participation, we purposely limited the number of questions on the survey and made responding easy with a multiple choice response format. Obviously, this diminished the richness of the data that were obtained from our sample of scientists. We were not able to examine the relative prevalence of other basic research aims such as description and replication that often drive investigations and more nuanced research strategies. Future research will need to take a much more open ended approach to ascertain the diverse goals and approaches characterizing the scientific enterprise.

Yet another limitation of our study is that self-reports were used to explicate the research goals and strategies of psychological scientists. People, of course, are not always aware of what they do or why they do what they do [20]. Moreover, self-reports may be biased by a host of factors including self-presentation concerns, acquiescence, reactance, and memory lapses [21]. As a consequence, there is often a significant gap between self-reports and actual behavior. Our concern is diminished by the belief that scientists tend to have a clear sense of their aims and approaches because of the frequency with which they are required to articulate their thoughts and activities in presentations, forums, articles, and grant proposals. Our confidence is also increased by the fact that the research approach reported by our sample of scientists was entirely consistent with the archival findings of Uchino, et al [4].

The sample in our study was far from representative of psychological scientists across the globe. The individuals who were solicited for participation were affiliated with six different research institutions exclusively in the United States, with only one developmental psychologist responding. Thus, caution should be exercised in generalizing to all psychologists.

Although our sample was limited to psychological scientists, the goals and strategies that were reported may be typical of all fields of science. We believe that in most scientific investigations, a confirmatory approach is used to test the non-absolute hypothesis that a phenomenon exists in some conditions. The approaches of other disciplines and the generality of our findings are topics that will need to be examined in future studies.

### The Limits of Falsification

Popper’s [1] logic of falsification was more than a prescription for how scientists should proceed. He regarded it as a description of how science actually works and progresses. However, numerous philosophers and scientists have argued that scientific theories are based more on corroborations than falsifications [3]. Moreover, disconfirmations are commonly discounted or dismissed because of possible “misassumptions” [22] such as insensitive measures, improper operationalizations, and weak manipulations. Even reliable falsifications rarely lead to the rejection of hypotheses. Instead, theories are typically modified to accommodate disconfirming findings [23, 24]. This was recognized by Popper [1] who believed that such ad-hoc adjustments were symptomatic of weak theory. In his view, the best (and most scientific) theories are those that are readily falsifiable. Because of the problems with simple or naïve falsification, he later proposed a more conventional and sophisticated form of “falsification” in which the weight of the evidence gathered by a field serves as the basis for the decision to reject or refute one theory in favor of another [1]. Nevertheless, he remained adamantly opposed to the idea of the confirmation of a theory throughout his career [25,26]. It is his conceptions of simple falsification that guide much of contemporary thinking in psychological science [27,28].

Our study adds to the literature on falsification by suggesting that the disconfirmation strategy once prescribed by Popper is actually normatively incorrect for the hypotheses that are most frequently investigated. In tests of the non-absolute hypotheses that dominate psychological science, falsifying evidence is relatively non-diagnostic. In contrast, the confirmatory approach that is typical of the field appears to be the most informative test of the non-absolute hypotheses that phenomena occur in some conditions.

Our analysis does not suggest that confirmation is more diagnostic than disconfirmation or vice versa; rather the informativeness of a search depends on the hypothesis under investigation. In this vein, it is worth noting that a confirmation of the non-absolute proposition that some instances are characterized by a test relation and a disconfirmation of the absolute proposition that no instances are characterized by a test relation are equivalent in their meaningfulness. Confirming that a relation sometimes exists by disconfirming the absolute proposition that a relation does not exist, of course, is precisely what is done in null hypothesis testing.

Our findings and analyses are largely mute regarding the controversy surrounding null hypothesis significance testing in psychological science. Our research does not speak to the criteria that should be used in tests or the statistical meaningfulness of the rejection of the null. Nevertheless, there is one issue that our study does address very sharply. Meehl [27] and later Dar [28] argue that null hypothesis testing falls short because confirmations are much less critical to theory development than the refutations afforded by other approaches. We believe that this particular criticism is misguided because of the limited diagnosticity of disconfirmations for tests of non-absolute hypotheses and because of the other problems associated with falsification discussed above.

#### Can Scientific Hypotheses be Verified?

One of the most influential ideas proposed by Popper [1] is that theories can never be conclusively verified. Our analysis suggests, quite fittingly, that this belief is correct sometimes. Universal generalizations can never be verified because an instance may be uncovered that is inconsistent with predictions. However, non-absolute hypotheses that a phenomenon occurs sometimes can be verified with a single confirming instance. The meaningfulness of such validation, of course, is more of a philosophical than an empirical question. Philosophers undoubtedly will have much to say about the informativeness (or uninformativeness) of confirmations of non-absolute hypotheses.

Although a study may confirm that a hypothesized relation sometimes exists, the results are specific to a particular time, context, sample, and set of procedures. Nevertheless, scientists assume that phenomena are relatively general and present in conditions beyond those examined in a study. After initial confirmation, they commonly investigate the scope of an observed relation. Although hypotheses can only be tentatively verified given the logical limitations of inductive inference, some philosophers have argued that each successive confirmation provides grounds for an increase in the probability of the hypothesis being “correct” [29–31]. Although intuitive, the probabilistic approach is primarily aimed at scoring the evidence for universal hypotheses and has met with both mathematical and philosophical problems [32].

An important category of non-absolute scientific generalizations which have been discussed extensively by philosophers are ceteris paribus laws [33–35]. These are laws that are presumed to hold true if there are no interferences or disturbing factors (ceteris paribus is Latin for "all other things being equal”). Many of the non-absolute hypotheses that are investigated in psychological science are not candidates for ceteris paribus laws because they are not presumed to be general. That is, the hypothesized relation is not expected to normally or typically occur in most instances. Often psychological scientists begin an investigation of a possible effect or relation without a clear sense of its scope. Following an initial demonstration, studies commonly attempt to determine not only the conditions in which a phenomenon is present but also the conditions in which it is absent. As our survey suggests, a disconfirmatory approach is much more frequent in the later stages of research. Often studies reveal that a phenomenon is limited largely to a narrow set of conditions. Some philosophers [36] distinguish “exclusive” ceteris paribus laws that refer specifically to effects or relations that occur only when particular factors are present.

Our findings and analysis suggest that science is a very different enterprise than that envisioned by Popper [1]. Research more often begins with tests of non-absolute hypotheses of limited scope than tests of universal laws. Because most hypotheses are non-absolute, they are not readily subject to the falsification that he initially postulated as the foundation of scientific advancement. Researchers much more commonly strive to confirm their ideas than falsify them. This approach is logically justifiable for tests of the non-absolute hypotheses that are investigated in most studies in psychological science. The ad hoc theorizing that Popper decried typifies science because theories are works in progress that are developed through the assimilation of new data.

## Supporting Information

S1 Dataset Survey Data. (XLS)
